# Effect of preparation size on the removal of accumulated hard-tissue debris from the mesial root canal system of mandibular molars using SWEEPS technology

**DOI:** 10.1007/s00784-023-04862-1

**Published:** 2023-01-16

**Authors:** Tina Rödig, Valerie Westbomke, Franziska Haupt, Marc Münster, Steffi Baxter

**Affiliations:** 1 Department of Preventive Dentistry, Periodontology and Cariology, University Medical Center Göttingen, Robert-Koch-Str. 40, 37075 Göttingen, Germany; 2 Endopur, Clinic for Endodontology, Frankfurt, Germany

**Keywords:** Hard-tissue debris, Mandibular molar, Micro-computed tomography, Photoacoustic streaming, Preparation size, SWEEPS

## Abstract

**Objectives:**

This study assessed the influence of preparation size on the efficacy of shock wave–enhanced emission photoacoustic streaming (SWEEPS) and conventional irrigation (CI) on removal of accumulated hard tissue debris (AHTD) from isthmus-containing mandibular molars using micro-computed tomographic analysis.

**Materials and methods:**

Sixty extracted mandibular molars with two mesial canals connected by an isthmus were selected. Canals were shaped with Mtwo instruments (VDW, Munich, Germany) up to sizes 25/.06, 40/.04 or 40/.06 (*n* = 20), and specimens were distributed into 2 final irrigation groups (*n* = 10): SWEEPS and CI. Roots were scanned at a resolution of 10.5 µm before and after preparation and final irrigation. Data sets were co-registered, and the percentage reduction of AHTD calculated for each specimen was statistically compared using analysis of variance with a of 5% significance level.

**Results:**

The preparation size did not significantly influence the percentage reduction of AHTD (*p* < 0.05), whereas the final irrigation technique had a significant effect on debris removal (*p* < 0.05). A significant reduction of AHTD was achieved after final irrigation in all groups (*p* < 0.05); however, SWEEPS was associated with a significantly greater percentage reduction of debris than CI (*p* < 0.05). None of the specimens presented a completely clean isthmus.

**Conclusions:**

Removal of AHTD was not significantly affected by the preparation size. SWEEPS was associated with significantly less debris than CI.

**Clinical relevance:**

SWEEPS performed significantly better than CI regarding the removal of AHTD from isthmus-containing mandibular molars irrespective of the preparation size.

## Introduction

Mechanical root canal preparation results in accumulated hard-tissue debris (AHTD) in anatomic complexities of the root canal system [1–3]. In infected root canals, AHTD may harbour microorganisms and serve as a reservoir for root canal reinfection. Additionally, debris packed in irregularities of the root canal space may impede thorough disinfection in cases with apical periodontitis [2, 4, 5]. It can also negatively interfere with the sealing ability of root canal filling materials as the remaining dentine debris hinders the gutta-percha to penetrate into recesses of the root canal space even when thermoplasticized obturation techniques are used [6] and prevent their adaptation into hard-to-reach areas [6, 7].

Conventional irrigation (CI) using a syringe and needle is often inefficient on the removal of AHTD from anatomic complexities because fluid penetration and exchange beyond the needle tip is limited [8]. Moreover, irrigant velocity and wall shear stress which are important parameters for the mechanical cleaning effect of irrigation [9, 10] are restricted during syringe irrigation thereby decreasing the penetration depth of the irrigation solution into anatomic irregularities such as fins or isthmuses [3, 7, 11, 12]. Thus, several activation techniques such as sonic or ultrasonic devices have been proposed to enhance the mechanical flushing action of the irrigant to increase its effectiveness [13, 14]. Recent studies have investigated the efficacy of these machine-assisted agitation systems in removing AHTD from isthmus-containing mesial root canals of mandibular molars [15–18]. Although sonically or ultrasonically activated irrigations are the predominant techniques in clinical application [19], laser-activated irrigation (LAI) has been introduced as a promising tool to improve root canal debridement [20–24]. The effect of LAI is based on the absorption of laser energy from erbium lasers (erbium-doped yttrium aluminium garnet, Er:YAG) in an aqueous irrigant [25] which creates vapour bubbles at the fibre tip. These vapour bubbles expand and collapse, resulting in cavitation-induced shock waves and microjets in the fluid [25]. However, these observations have been mainly noted in large liquid reservoirs and cannot be directly applicable to phenomena inside the confined volume of a root canal system [26, 27]. It was recently shown that single laser pulses did not result in the emission of shock waves in narrow reservoirs such as liquid-filled root canals [26]. The main reasons for this decelerated cavitation bubble dynamics are the friction on the root canal walls and the limited space available for a quick displacement of the irrigant during the expansion and contraction of the bubble [27, 28].

Photon-induced photoacoustic streaming (PIPS) uses Er:YAG laser energy (2940 nm) at subablative power levels (0.3 W, 20 mJ at 15 Hz) and a single short pulse duration (50 µs) [24, 29] and has been reported to be more effective than syringe irrigation in removing debris from root canal irregularities [30–33].

Recently, a novel shock wave–enhanced emission photoacoustic streaming (SWEEPS) modality for Er:YAG laser has been proposed, which is based on the delivery of pairs of laser pulses, properly timed to generate enhanced irrigant streaming and shock wave emission [27, 34]. During the SWEEPS mode, a fibre tip is placed inside the pulp chamber, which emits a subsequent laser pulse into the irrigant when the initial bubble is in the final phase of its collapse [27]. The secondary bubble exerts pressure on the collapsing initial bubble, thus accelerating its collapse and resulting in the formation of shock waves and an improved photoacoustic current, thereby increasing the cleaning and antimicrobial efficacy of the standard PIPS procedure [27]. This phenomenon has been observed even in spatially confined root canals, as enhanced shock waves were created using the SWEEPS modality [27].

Until now, limited data is available for the SWEEPS technique with regard to the removal of dentine debris. A recent study using micro-computed tomography (micro-CT) to assess the removal of AHTD from the root canal system of mandibular molars reported significantly less debris after SWEEPS activation compared to PIPS and ultrasonically activated irrigation [35].

As opposed to the conventional LAI procedure, in which root canal enlargement to a size #30 is mandatory to allow the laser fibre to be inserted in the apical third of the root canal [36, 37], PIPS and SWEEPS techniques require the fibre tip to be placed into the irrigant reservoir in the pulp chamber. Thus, some authors suggested that a minimally invasive root canal preparation to apical sizes 20/0.06, 25/0.06 or 20/0.07 is considered sufficient for effective debridement [24, 29]. Although this concept aims to preserve a maximum amount of healthy dental hard tissues in order to maintain the structural integrity of the tooth [38, 39], irrigant flow and penetration is compromised in the apical root canal third during syringe irrigation due to the limited insertion depth of the irrigation needle and the low wall shear stress [9, 40, 41].

Previous studies investigating the debridement and cleaning efficacy of SWEEPS selected different apical preparation sizes ranging from 25/0.04 to 40/0.06 [35, 42–45]. However, these studies can only be compared to a limited extent since apical preparation size and taper affect flushing action, wall shear stress and irrigant replacement during syringe and ultrasonically activated irrigation [9, 40, 46–49]. Moreover, it was shown that sequential apical enlargement from sizes 25 to 40 significantly reduced the amount of accumulated debris using a conventional irrigation protocol [50]. Thus, it can be assumed that root canal dimensions also influence the performance of the novel SWEEPS technique.

So far, there is no information on the effect of preparation size in the removal of debris using SWEEPS. Thus, the aim of this in vitro study was to evaluate the influence of preparation size and taper on the efficacy of the SWEEPS technique and conventional syringe irrigation in removing AHTD from the isthmus of the mesial canals of mandibular molars using micro-CT imaging. The null hypotheses tested were that neither the apical preparation size nor the final irrigation protocol has an influence on the reduction of AHTD.

## Material and methods

### Sample size estimation

Sample size calculation was based on a previous study on the removal of hard-tissue debris with four final irrigation protocols [11]. In that study, the percentage volume of AHTD after passive ultrasonic irrigation was 0.6% ± 0.7% compared with apical positive pressure (3.7% ± 1.5). An alpha-type error of 0.05, power beta of 0.95% and allocation ratio N2/N1 of 1 was also specified. A total of 7 samples per group were calculated as the minimum size for observing significant differences (https://www.stat.ubc.ca/~rollin/stats/ssize/n2.html).

### Specimen selection

After approval of the local Ethics committee (protocol no. 27/8/13), a total of 241 mesial roots of mandibular molars with fully developed apices and without previous endodontic treatment were selected and stored in 0.1% thymol solution until use. Mesial canals were accessed, and a size 10 reamer (Dentsply Sirona, Ballaigues, Switzerland) was inserted into the root canal until the tip of the instrument was just visible at the apical foramen. Digital radiographs were taken in a bucco-lingual direction, and root canal curvatures and radii were measured [51, 52]. Only specimens with curved root canals ranging from 20 to 40° and a radius between 5.5 and 16.5 mm were included. The roots were pre-scanned in a micro-CT device (SkyScan 1272; Bruker-microCT, Kontich, Belgium) with an isotropic resolution of 21.7 µm to obtain a preoperative outline of root canal anatomy. Overall, 60 specimens presenting a type II Vertucci’s canal configuration system [53] with two mesial root canals containing an isthmus with a length of at least 2 mm in the corono-apical dimension [54] were obtained.

The apices of the roots were sealed with fast-set epoxy resin to create a closed-end system, and the reconstruction of the distal wall of the pulp chamber was performed using a dentine adhesive and a resin composite to provide a reservoir for the irrigant. The cusps of the specimens were flattened to a standardized root length of 19 mm with a working length (WL) of 18 mm. Subsequently, the roots were scanned with a high isotropic resolution of 10.5 µm at 80 kV, 125 µA, 180° rotation around the vertical axis, a rotation step of 0.4°, a camera exposure time of 3230 ms and frame averaging of 3. X-rays were filtered using a 1-mm-thick aluminium filter. The acquired projection images were reconstructed (NRecon v.1.7.0.3 software, Bruker-microCT) using 45% beam hardening correction and ring artefact correction of 20–22. Volumetric analyses of the 3-dimensional models were performed by using CTAn v.1.17.7.2 software (Bruker-microCT). Subsequently, the specimens were allocated to three groups (*n* = 20) based on the morphological parameters of the root canal system (root canal volume and surface, isthmus length in corono-apical direction and isthmus width in bucco-lingual direction). The homogeneity of the values among the groups was verified using Kruskal–Wallis test, thus confirming anatomical matching between experimental groups (*p* > 0.05; Table 1).

**Table 1:** Intracanal parameters of sixty mesial roots anatomically matched in three groups with different apical preparation sizes (means and SD). Debris volume (mm^3^) before and after final irrigation and percentage reduction (%) of AHTD (means and SD)

Preparation size	**25/.06**		**40/.04**		**40/.06**	
Final irrigation technique	CI	SWEEPS	CI	SWEEPS	CI	SWEEPS
	Mean ± SD		Mean ± SD		Mean ± SD	
Before preparation						
Root canal volume (mm^3^)	4.61 ± 2.72		4.33 ± 1.81		4.17 ± 1.53	
Root canal surface (mm^2^)	115.96 ± 65.04		106.70 ± 40.07		97.61 ± 34.75	
Isthmus length (mm)	3.99 ± 2.50		3.86 ± 1.95		3.54 ± 1.80	
Isthmus width (mm)	2.02 ± 0.63		1.95 ± 0.50		1.80 ± 0.38	
After preparation						
Root canal volume (mm^3^)	9.62 ± 3.00	9.91 ± 4.92	9.53 ± 2.13	10.78 ± 2.20	10.41 ± 2.58	12.01 ± 4.06
Root canal surface (mm^2^)	150.15 ± 57.02	167.95 ± 121.60	135.11 ± 53.22	179.27 ± 85.19	106.07 ± 32.31	130.34 ± 48.03
Unprepared area (%)	48.35 ± 17.69	41.86 ± 18.08	58.94 ± 15.87	56.45 ± 15.64	41.32 ± 24.22	37.84 ± 15.39
Debris (mm^3^)	1.38 ± 0.86^A^	1.65 ± 1.15^A^	1.64 ± 0.68^A^	1.81 ± 0.88^A^	1.37 ± 0.62^A^	1.54 ± 0.82^A^
After irrigation						
Debris (mm^3^)	0.96 ± 0.83^B^	0.65 ± 0.32^B^	1.00 ± 0.54^B^	0.60 ± 0.38^B^	1.03 ± 0.45^B^	0.51 ± 0.35^B^
Reduction (%)	32.0 ± 22.3^a^	52.7 ± 28.2^b^	37.7 ± 23.9^a^	62.8 ± 20.8^b^	23.5 ± 16.9^a^	65.3 ± 20.7^b^

Different uppercase superscripts indicate significant differences between debris volumes before and after final irrigation within subgroups (*p* < 0.05)

Different lowercase superscripts indicate significant differences between irrigation techniques within the same preparation size (*p* < 0.05)

*AHTD* accumulated hard-tissue debris, *CI* conventional irrigation, *SWEEPS* shock wave enhanced emission photoacoustic streaming

### Root canal preparation

A glide path was established with a size 10 reamer (VDW, Munich, Germany) up to WL. Both mesial root canals were instrumented using the basic sequence of Mtwo instruments (VDW) 10/0.04, 15/0.05, 20/0.06 and 25/0.06 taper driven with an electric motor (VDW Silver, VDW). According to the manufacturer, all instruments were used with a brushing motion until WL was reached. Each time the instrument was removed, 2 mL NaOCl (1%) was applied using a 30-gauge open-end tip needle (Endo-EZE, Ultradent, South Jordan, UT, USA). According to the experimental groups, no further root canal preparation was performed in group 1 (25/0.06). The root canals of the remaining specimens were further enlarged using the instrument sequence recommended by the manufacturer, followed by an additional rinse with 2 mL NaOCl (1%) after each NiTi instrument, resulting in either a final apical size 40/0.04 (group 2) or 40/0.06 (group 3). Instruments were discarded after four uses or if an unwinding occurred. After completion of preparation, an additional rinse with 2 mL NaOCl (1%) was performed, root canals were dried with paper points and a second micro-CT scan was performed and reconstructed using the same parameters as the first scan.

Postoperative scans were co-registered with their respective preoperative data sets in DataViewer 1.5.2.4 software (Bruker-microCT) using a pseudo 3D registration tool. Quantification of AHTD was performed by the difference between the root canal spaces before and after preparation using CTAn software (CTAn, 1.17.7.2, Bruker-microCT). The volume of interest (VOI) was defined as the region previously occupied by air in the non-prepared root canal. The additional material that was pushed into the isthmus during preparation (before final irrigation procedure [*V*_BFI_]), with density similar to dentine, was considered as debris, and its volume was estimated as the volume of dentine-like material inside the VOI [2, 55]. In addition, unprepared areas were quantified by calculating the number of static surface voxels, which was expressed as a percentage of the total number of surface voxels.

### Final irrigation protocols

Specimens from each experimental group were further assigned into two subgroups (*n* = 10) according to the final irrigation technique used: CI and SWEEPS. The equality of the values regarding the root canal volumes and surfaces after preparation and the percentages of unprepared areas as well as the volume of AHTD before final irrigation among the subgroups was evaluated using Mann–Whitney *U*-test. *p*-values were adjusted using Bonferroni-Holm method (*p* > 0.05) (Table 1).

Specimens of each group were submitted to a final rinse using 15 mL of 1% NaOCl, 10 mL distilled water and 5 mL of 17% EDTA during a total time of 4.5 min according to one of the following irrigation protocols:

#### CI

Five millilitres of NaOCl was applied for 30 s with a syringe and a 30-gauge Endo-EZE needle (Ultradent) positioned at 2 mm from WL, followed by a 30 s resting phase. After this procedure has been repeated three times, root canals were irrigated for 30 s with 5 mL distilled water, for 30 s with 5 mL EDTA and for 30 s with 5 mL distilled water.

#### SWEEPS

Five millilitres of NaOCl was continuously delivered using a syringe and a 30-gauge Endo-EZE needle (Ultradent) to maintain hydration during activation. A special Auto SWEEPS Er:YAG laser modality and a special conical fibre tip with a flat end (SWEEPS 600, Fotona) were used at a wavelength of 2940 nm Er:YAG laser at 0.6 W, 15 Hz and 20 mJ per pulse without water and air. The SWEEPS tip was inserted 3 mm deep into the access cavity and activated for 30 s, followed by a 30 s resting phase between activation when using NaOCl. This procedure was repeated three times. Then, 5 mL distilled water was continuously irrigated and activated for 30 s, followed by a continuous flush of 5 mL EDTA within an activation time of 30 s. Finally, 5 mL distilled water was flushed and activated for 30 s as previously described.

Final irrigation procedures were performed by one operator. Root canals were dried with sterile paper points, and a final micro-CT scan was performed. Data sets of the samples after final irrigation were co-registered with their respective counterparts after preparation, and the volume of AHTD (mm^3^) in each root canal system was calculated (*V*_AFI_). The percentage reduction was obtained according to the following formula [(*V*_BFI_ – *V*_AFI_)/*V*_BFI_]*100, where *V*_BFI_ and *V*_AFI_ are the volume of AHTD (mm^3^) before and after final irrigation procedures, respectively.

### Statistical analysis

As normality assumptions were verified in the data set, means were compared using a two-way ANOVA to calculate the percentage reduction of debris after final irrigation. Paired sample *t*-tests were carried out to analyse differences between debris volumes before and after final irrigation. *p*-values were adjusted using Bonferroni-Holm correction method. The level of statistical significance was set at *α* = 0.05. All analyses were performed with Statistica software v. 13.0 (StatSoft, Tulsa, OK, USA).

## Results

Root canal volumes, surface areas, percentages of unprepared areas and volumes of AHTD after preparation did not differ significantly between the two tested groups within the same preparation size (*p* > 0.05; Table 1).

The preparation size did not significantly influence the percentage reduction of AHTD (*p* > 0.05); thus, the first null hypothesis was accepted.

Conversely, the final irrigation technique significantly affected the percentage reduction of AHTD (*p* < 0.05; Table 1). Final irrigation using SWEEPS was associated with a significantly greater percentage reduction of debris than CI (*p* < 0.05; Table 1). Therefore, the second null hypothesis was rejected. No interaction of factors (preparation size, final irrigation technique) was observed (*p* > 0.05).

Nevertheless, the percentage volume of AHTD after preparation was significantly reduced irrespective of the final irrigation technique (*p* < 0.05).

**Fig. 1:**
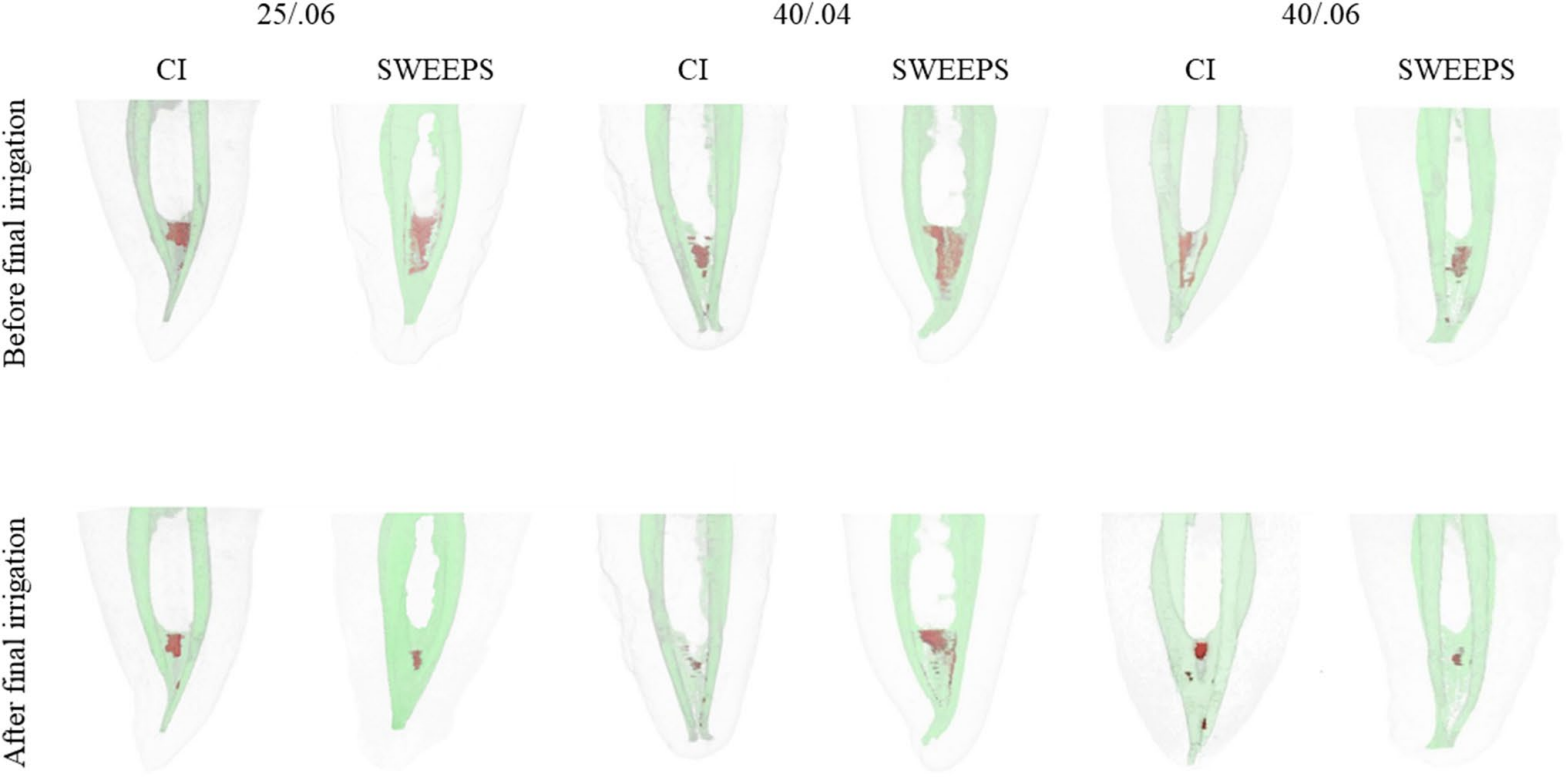
Representative 3D reconstructions of the mesial root canal systems before and after final irrigation with conventional syringe irrigation (CI) and shock wave–enhanced emission photoacoustic streaming (SWEEPS). Accumulated hard-tissue debris (AHTD) is depicted in red

None of the specimens presented a completely clean isthmus. Three-dimensional models of representative mesial root canal systems in each group demonstrate the distribution of AHTD after preparation and the remaining debris after final irrigation (Fig. 1).

## Discussion

This in vitro study assessed the effectiveness of laser-activated and conventional final irrigation on the removal of AHTD from mesial root canal systems of mandibular molars prepared to different apical sizes using micro-CT imaging. In laboratory-based research, the removal of accumulated dentine debris from root canals is usually used as a surrogate end-point to evaluate the efficacy of irrigation [56]. Although recent research demonstrated the visualization of pulp tissue to assess root canal debridement by using contrast-enhanced micro-CT imaging [57], the vast majority of investigations has applied conventional micro-CT technology as a precise tool for quantitative and qualitative evaluation of hard-tissue debris within the root canal system [2, 16, 18, 35, 55, 58, 59]. The main advantage of this three-dimensional, non-destructive technology is considered to be the longitudinal evaluation of the same specimen at different time-points during several experimental procedures [18, 35]. Nevertheless, micro-CT imaging enables only in vitro measurements due to the high radiation and long scanning times [55].

Isthmus-containing mesial roots of mandibular molars were selected for the present study as these teeth exhibit a complex internal anatomy [60] where hard-tissue debris tends to accumulate during instrumentation [2]. These isthmuses represent a major challenge for adequate cleaning and disinfection [61] since effective delivery and exchange of irrigants into these hard-to-reach areas of the root canal system is often impeded [15, 62].

In order to increase the internal validity of the study by substantially reducing the anatomical bias among the specimens, groups were balanced with regard to morphometric parameters of the root canal systems (volume, surface area, length and width of isthmus) [12, 63, 64]. Additionally, root canal volumes and surface areas after preparation as well as the percentages of non-instrumented surface areas did not differ significantly between CI and SWEEPS within the same preparation size. Although the root canals were prepared to different apical sizes, equal amounts of AHTD were obtained during instrumentation in all groups prior to final irrigation. As a result, statistical analysis demonstrated no significant differences between experimental groups and confirmed a consistent baseline with respect to relevant parameters prior to final irrigation.

After separation of the mesial roots, the distal wall of the pulp chamber was reconstructed with a resin composite to provide a reservoir for the irrigant [17, 62] as suggested for laser-activated irrigation protocols [24]. Subsequently, the length of the specimens was standardized to 19 mm by flattening of the cusps, resulting in slightly different volumes of the irrigant reservoirs. In previous studies evaluating the efficacy of laser-activated irrigation using PIPS and SWEEPS, the specimens were also reduced to a standardized length of 12 mm [44] or 14 mm, and an artificial irrigant reservoir was created with Gates-Glidden drills [24]. Although the volume of the irrigant reservoir was not standardized in the present study, the irrigant in the access cavity was continuously replenished to maintain a constant irrigant level which is crucial for the success of laser-activated irrigation techniques [21]. Conversely, even if the crowns of the specimens remained intact in previously published studies on LAI [35, 59], this does not necessarily indicate standardized volumes of the access cavities nor the irrigant reservoirs.

Previous studies on AHTD removal used none or minimal irrigation solution during instrumentation to obtain a maximum amount of accumulated debris in the isthmus area [2, 11, 15, 35]. In contrast, NaOCl was used during root canal preparation in the present study as it is not advisable to modify the chemomechanical preparation protocols to favour debris accumulation since this creates an unrealistic challenge for the irrigants [56]. In the present study, the mean debris volume related to the total root canal system before preparation was 39.9% ± 11.4%. In a previous investigation, a mean debris volume of 29.2% ± 14.5% was created during instrumentation with ProTaper instruments to size F3 although no irrigation was performed [2]. These discrepancies between preoperative amounts of debris are notable and can be explained with differences in sample selection with regard to Vertucci’s configuration presenting small or large isthmus areas. Moreover, quantitative data extracted from micro-CT scans are strongly affected by the voxel size [56, 65]. In the present study, a voxel size of 10.5 µm was applied during micro-CT imaging which is within the range between 8.6 µm and 30 µm reported previously [3, 11, 16, 18, 35, 59]. Another confounding factor which contributes to variances regarding the preoperative amount of AHTD among previous studies is related to different calculations as the volume of AHTD can be expressed as the percentage of the total root canal system volume before [2, 3, 17] or after preparation [11, 15, 18, 35, 59]. For example, in previous micro-CT studies, substantially smaller amounts of debris between 7 and 15% with ProTaper Universal F3 and WaveOne Gold Primary instruments were produced, respectively, despite minimal or none irrigation [15, 35]. Nevertheless, these debris volumes were related to the total root canal system after preparation which impedes a direct comparison of these findings. These methodological differences highlight the relevance of standardizing experimental protocols to allow a more precise analysis of the various irrigation techniques applied [66].

In the present study, the novel SWEEPS technique was compared to conventional syringe irrigation as this procedure remains the most popular technique for delivering root canal irrigants amongst endodontists and general dentists [19, 67, 68] to set a common point of reference [56]. This approach facilitates a realistic comparison between any irrigant activation technique and the traditional irrigation protocol [66].

Irrespective of the apical preparation size, SWEEPS resulted in a significantly higher percentage reduction of AHTD than conventional manual irrigation. This increased efficiency of SWEEPS has been explained by the photoacoustic shock waves and cavitation generated by the dual pulsed laser [27, 34, 69], which improved the removal of a greater amount of dentine debris compared with syringe irrigation. Previous studies evaluating the efficacy of PIPS on the removal of debris from the mesial root canal system of mandibular molars [32] or from artificial root canal irregularities in canine roots [31] also reported advantages over the conventional syringe irrigation, supporting the findings of the present study.

Instrumentation size did not significantly influence the removal of AHTD in the present study. This result is in contrast to a previous micro-CT investigation in which root canal enlargement from size 25 to size 40 significantly reduced the overall amount of hard tissue debris by 34% [50]. However, this study focused on the amount of AHTD produced after chemomechanical preparation to different apical sizes with single-file reciprocating systems and a conventional multifile rotary system and not on the final irrigation procedure [50]. These different findings may be explained by anatomical variations such as isthmus width and length which may affect irrigant penetration and the amount of debris removal. A recent study on biofilm removal using confocal microscopic imaging demonstrated the challenges of debriding the isthmus region and the influence of the cross-sectional shape of the isthmus, with the most restricted isthmus area being the most difficult to clean [69]. Further reasons for the divergent results with regard to the reduction of hard-tissue debris by apical enlargement may be differences in irrigant volume and concentration, flow rate and irrigation time.

Final irrigation using conventional irrigation significantly reduced the amount of AHTD created by chemomechanical preparation which is consistent with a previous micro-CT study with a similar experimental set-up [17]. Syringe irrigation was performed according to an optimum clinically relevant protocol as several factors such as needle type, insertion depth and flow rate were shown to influence efficacy of manual irrigation [8, 9]. An open-ended needle was used for irrigant delivery because the high-velocity jets created by this needle are more effective in terms of irrigant penetration and exchange [9, 70]. The applied flow rate of 0.166 mL^−1^ represents an intermediate value in the range employed by clinicians and can be achieved clinically [71]. A recent study using a computational fluid dynamics model reported a higher irrigant velocity and wall shear stress in a simulated isthmus when the flow rate was increased from 0.033 to 0.166 mL^−1^ [72]. Another factor that promoted removal of AHTD by syringe irrigation was the needle depth placement at 2 mm short of WL. Positioning the irrigation needle at WL minus 1 mm resulted in percentage levels of AHTD removal from the isthmus of mandibular molars almost three times higher than an insertion depth of 5 mm from the WL [73].

Previous studies on AHTD removal from isthmus-containing mandibular molars using micro-CT imaging reported a higher reduction of AHTD after syringe irrigation between 43.7 and 57.1% [11, 16, 74]. This difference may be attributed to anatomical variations such as C-shaped root canals [74] or a Vertucci type I configuration [11] which may allow better penetration of the irrigants into the isthmus area [62]. In addition, flow rates and needle insertion depth for syringe irrigation, overall irrigant volume and volume of AHTD after preparation varied significantly between these studies and therefore might have influenced these results.

To date, only one study has investigated SWEEPS for removing AHTD from the mesial root canal system of mandibular molars using micro-CT imaging, which reported an overall debris reduction of 84.3% [35]. Although these findings for SWEEPS were better than in the present study, a reliable comparison of these data is hardly feasible due to several differences in the experimental set-up. Firstly, anatomical variations such as the dimensions of root canals and isthmuses have an impact on AHTD removal as penetration of the irrigant is reduced in narrow isthmuses [62]. To overcome anatomical variations, 3D-printed root canal models were recently introduced to allow a reproducible and standardized assessment of different irrigation techniques [72, 75]. However, substitution of dentine by artificial root canals may also affect irrigant penetration since the surface properties of dentine are different from those of hydrophobic resins [56, 76]. Secondly, different final irrigation protocols were applied for SWEEPS with regard to the additional use of EDTA or the resting time of the irrigant between activation. Thirdly, Yang et al. (2020) did not include conventional syringe irrigation as a control group which impedes a comparison of the SWEEPS technique to a current clinical standard [56, 66].

None of the irrigation protocols was able to render the mesial root canal system free from dentine debris which is in accordance to several previous studies [3, 11, 12, 16–18, 50, 77].

Histological analysis of mesial root canal systems of mandibular molars revealed that biofilms embedded in hard-tissue debris remain in inaccessible recesses and isthmuses after root canal preparation [4]. Moreover, a percentage of non-instrumented root canal surfaces ranging from 37.84 to 58.94% emphasizes the suboptimal mechanical action of Mtwo instruments on canal walls. Nevertheless, previous micro-CT studies also reported a range of 2.66 to 79.04% untouched root canal walls after biomechanical preparation irrespective of the instrumentation technique [78–81]. In infected root canals, these unprepared surfaces contain pulp remnants and bacteria [5] which are suggested to be the primary cause for endodontic treatment failure [82]. Thus, the need to optimize chemomechanical preparation to reduce the accumulation of hard-tissue debris is highlighted [15, 50].

A limitation of the present study is that AHTD was used as a surrogate end-point to evaluate the efficacy of the irrigation techniques under investigation since this parameter has not been directly correlated to the healing of apical periodontitis [56]. Thus, future studies on root canal irrigation should focus on the validation of this surrogate end-point whether it is suited to be correlated with the reduction of the microbial load.

## Conclusions

The preparation size did not significantly affect the removal of AHTD from isthmus-containing mandibular molars. Although both final irrigation techniques resulted in a significant reduction of AHTD, SWEEPS performed significantly better than conventional irrigation.
